# Management of Peptic Ulcer Bleeding in Different Case Volume Workplaces: Results of a Nationwide Inquiry in Hungary

**DOI:** 10.1155/2012/956434

**Published:** 2012-09-05

**Authors:** István Rácz, Tibor Kárász, Krisztina Lukács, Ferenc Rácz, János Kersák, Judit Wacha, Tibor Szalóki, Magdolna Szász, István Gyenes, István Altorjay

**Affiliations:** ^1^Division of Gastroenterology, Department of Internal Medicine, Petz Aladár County and Teaching Hospital, Győr 9024, Hungary; ^2^Department of Gastroenterology, School of Medicine, Debrecen University, Debrecen 4032, Hungary; ^3^Department of Internal Medicine, Jósa András County Hospital, Nyíregyháza 4400, Hungary; ^4^Department of Internal Medicine, Municipal Hospital, Siófok 8600, Hungary; ^5^Department of Surgery, Semmelweis University, Budapest 1083, Hungary; ^6^Department of Internal Medicine, Jávorszky Ödön Municipal Hospital, Vác 2600, Hungary; ^7^Department of Internal Medicine, Kenézy Gyula County Hospital, Debrecen 4031, Hungary

## Abstract

The aim of this study was to conduct a national survey to evaluate the recent endoscopic treatment and drug therapy of peptic ulcer bleeding (PUB) patients and to compare practices in high and low case volume Hungarian workplaces. A total of 62 gastroenterology units participated in the six-month study. A total of 3033 PUB cases and a mean of 8.15 ± 3.9 PUB cases per month per unit were reported. In the 23 high case volume units (HCV), there was a mean of 12.9 ± 5.4 PUB cases/month, whereas in the 39 low case volume units (LCV), a mean of 5.3 ± 2.9 PUB cases/month were treated during the study period. In HCV units, endoscopic therapies for Forrest Ia, Ib, and IIa ulcers were significantly more often used than in LCV units (86% versus 68%; *P* = 0.001). Among patients with stigmata of recent haemorrhage (Forrest I, II), bolus + continuous infusion PPI was given significantly more frequently in HCV than in LCV units (49.6% versus 33.2%; *P* = 0.001). Mortality in HCV units was less than in LCV units (2.7% versus 4.3%; *P* = 0.023). The penetration of evidence-based recommendations for PUB management is stronger in HCV units resulting lower mortality.

## 1. Introduction

Acute upper gastrointestinal bleeding (UGIB) is a common medical emergency situation. Peptic ulcer bleeding (PUB) is responsible for almost half of the cases of UGIB [1, 2]. Despite advances in diagnosis and treatment during the recent years, rebleeding occurs in about 10–30% after primary haemostasis, and the mortality is still around 5–10% [1–5]. The appropriate management for patients with acute gastroduodenal ulcer bleeding has been established over the last two decades in a number of randomised controlled trials and in several guidelines [6–13]. Recommendations for the management of PUB were also published in Hungary [14]. The most important elements of these recommendations were to organize and maintain a 24-hour emergency endoscopy services for UGIB patients, to use the Forrest classificaton for PUB patients, endoscopic haemostatic therapy preferably with a combined methods obligatory in cases with active bleeding, and strongly recommended in ulcer cases with visible vessels and also with adherent clots. Acid-suppressant therapy was recommended by i.v. proton-pump inhibitors following endoscopic haemostasis for 72 hours in ulcer cases with stigmata of recent haemorrhage.

The primary aims of our work were to conduct a national survey to evaluate the use of recommendations and guidelines in the daily routine management of PUB and also to compare practices and patient outcome data in high and low case volume workplaces.

## 2. Material and Methods

The survey was designed to evaluate the different steps of the management procedure of PUB. Additionally, some basic patient outcome data were also collected (Table 1).

A database of all Hungarian gastroenterology departments performing endoscopy and treating acute GI bleeding was available on the basis of the address list of the Endoscopy section of the Hungarian Society of Gastroenterology. The questionnaires were distributed and collected monthly by specially trained research assistants in those 62 gastroenterological workplaces (GI units) that responded positively to participate in the study. These 62 GI units from 39 cities account for 71% of the GI workplaces existing in Hungary. To ensure the validity of the collected data, the research assistants with the participation of the local study coordinator endoscopists reviewed and monitored each month all endoscopy reports and patient files of the endoscopy units searching for all documented data of UGIB patients. The aim of this rigorous data monitoring protocol was to collect reasonably high-quality data of current practices. All data were entered electronically and downloaded into a central repository on a monthly basis. In Hungary, only gastroenterologists or surgeons, when they have taken at least a two-year endoscopy training course, may perform upper or lower endoscopy.

### 2.1. Statistical Analysis

Data were collected and analysed using the statistical package SPSS version 11.0 (SPSS In. Chicago, IL, USA). Descriptive statistics were used to analyse and report the data. The chi-squared and the Fisher tests were used to determine differences between low and high case volume GI units and also for the analysis of two and multidimensional contingency tables. Multiple logistic regression was applied to evaluate the independent relations of selected factors for the use of combined haemostatic methods followed by bolus plus continuous infusion of PPI. The significance threshold was set at *P* < 0.05.

## 3. Results

### 3.1. General Data and Case Volume Differences

A total of 62 GI units provided data by completing the questionnaires in a six-month period during 2009 and 2010. A 24-hour emergency endoscopy service was guaranteed in 90% (*n* = 54) of the workplaces, and specialised endoscopy nurses were available in 85% (*n* = 51) for 24 hours. A total of 6,473 acute upper GI bleedings including 3,033 (46.9%) PUB cases were reported. Of the responded PUB cases, 89.2% were managed by gastroenterologists and 10.8% by surgeons.

A mean of 17.4 ± 8.2 UGIB and 8.15 ± 3.9 PUB cases per month per unit were reported, respectively. There were 23 units that reported more than 8.15 PUB cases per month, and the remaining 39 units had fewer PUB cases monthly than this mean. This selection offered the possibility to divide the reporting GI units into high case volume (HCV) and low case volume (LCV) units according to the monthly mean of ulcer bleeders of which they took care. In the 23 HCV units, a total of 1,789 PUB cases (mean 12.9 ± 5.4 cases/month), whereas in the 39 LCV units, a total of 1,244 PUB cases (mean: 5.3 ± 2.9 cases/month) were managed during the study period. These data reflect that the HCV units had more than twice as many PUB cases and experiences per month compared to the LCV workplaces.

**Figure 1:**
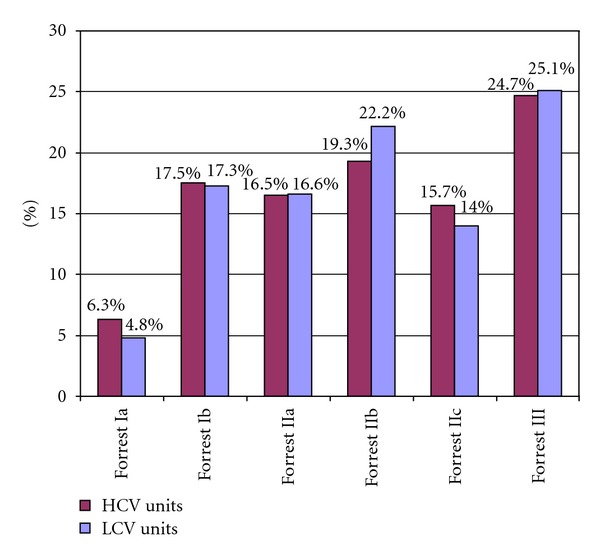
Proportion of bleeding ulcers according to Forrest classification in high case volume (HCV) and low case volume (LCV) units.

The Forrest classification was uniformly used both in HCV and LCV units. The proportion of emergency endoscopy findings according to the Forrest classification were similar comparing results obtained from HCV and LCV workplaces (Figure 1). The ratio of high-risk lesion bleeders (Forrest Ia-IIa) was also similar (*n* = 717; 40%) in HCV and LCV (*n* = 479; 38.5%) units. 

### 3.2. Endoscopic Haemostatic Therapy

Endoscopic haemostatic therapy was given for ulcers with spurting bleeding (Forrest Ia), oozing bleeding (Ib), nonbleeding visible vessels (IIa), adherent clot (IIb), black haematin-covered ulcer base (IIc), and clean ulcer base (III) in, respectively, 94%, 83%, 69%, 43%, 15%, and 4% of all responded PUB cases. In HCV units, endoscopic therapy was significantly more often used in Forrest Ia, Ib and Forrest IIa cases (*n* = 613; 85.4%) compared with similar Forrest grade cases in LCV units (*n* = 327; 68.2%) (Table 2).

The most frequently used haemostatic treatment modality either in mono or in combination was injection with diluted (1 : 10.000) epinephrine (*n* = 1108; 92.6%). As a combination treatment generally, injection and haemoclips or injection and coagulation were combined. For high-risk ulcers (Forrest Ia, Ib, IIa), the combined haemostatic attempts were significantly more frequently used in HCV than in LCV units (*n* = 207; 34% versus *n* = 61; 19%; *P* < 0.01), whereas the opposite result was detected regarding injection monotherapies, which were significantly more often used in LCV than in HCV units (*n* = 311; 65% versus *n* = 285; 46%; *P* < 0.001) (Table 2).

### 3.3. Acid-Suppressant Therapy

In our questionnaire we asked only for the postendoscopy acid-suppressive drugs because the preendoscopy i.v. PPI treatment policy was not yet established at the time of the survey.

Acid-suppressive drugs following the endoscopy were administered intravenously (i.v.) in the majority of PUB cases (*n* = 2516; 83.0%), mostly using PPI (*n* = 2425; 79.9%) and only seldomly using H_2_-receptor antagonists (*n* = 91; 3.0%). In less than half of all PUB cases (*n* = 1095; 45.2%), i.v. PPI was given in standard dosages twice or three times daily, whereas bolus PPI followed by a continuous infusion of 8 mg PPI per hour was used slightly more often (*n* = 1301; 53.6%). Among patients with stigmata of recent haemorrhage (Forrest I-II), bolus + continuous infusion PPI was given significantly more frequently in HCV than in LCV units (*n* = 888; 49.6% versus *n* = 413; 33.2%; *P* < 0.001) (Table 2).

According to multivariate analysis, the monthly PUB case volume of units was the only significant predictor factor for the use of best evidence combined endoscopic haemostasis followed by bolus plus infusion PPI in high-risk ulcers (Table 3).

### 3.4. Clinical Outcome

Overall rebleeding rates were comparable in HCV (*n* = 179; 10.1%) and in LCV (*n* = 118; 9.5%) units. Also rebleeding rates in high-risk ulcers (Forrest Ia-IIa) were similar in HCV (*n* = 141; 19.7%) and in LCV (*n* = 63; 19%) units.

Because of persistent bleeding or endoscopically untreatable severe rebleeding, surgery was needed slightly more frequently in LCV (*n* = 79; 6.4%) than in HCV (*n* = 92; 5.1%) units. In cases with endoscopically untreatable spurting bleedings, an immediate surgery was significanly (*P* < 0.05) more often warranted in LCV (*n* = 29; 49.2%) compared to HCV (*n* = 36;  32.1%) units.

The bleeding-related mortality rate of all PUB patients was 3.3% (*n* = 101). Mortality in HCV units (*n* = 48; 2.7%) was significantly (*P* = 0.023) less than the mortality in LCV units (*n* = 53; 4.3%). Those patients with initial spurting (Forrest Ia) and oozing (Forrest Ib) bleedings had higher mortality rate (*n* = 21; 7.6%) in the LCV than in HCV units (*n* = 29; 6.8%), but this difference was not significant (Table 2).

## 4. Discussion

This is the first clinical inquiry conducted in Hungary illustrating the daily routine endoscopic and pharmacological management of PUB. Similar surveys were performed previously in the Netherlands, France, and recently in Germany with the response rates of 73%, 34%, and 49%, respectively, positioning our survey with a 71% response rate in between these other surveys [15–17]. 

In the present study, unlike the previous surveys, epidemiological and practice differences of high case volume and low case volume units were compared as a primary aim. Nevertheless, the high and low volumes of endoscopy units were not a priori and numerically defined in the questionnaires but just based on the collected data reflecting mean number of PUB cases per month per units. This comparison was rational because in HCV units, more than twice as many PUB cases per month were treated compared to the LCV units; one may suspect that the differing volumes would alter the daily practices. 

In our study, entirely emergency or early endoscopy cases with endoscopic procedures within the first 24 hours after the admissions were reported [18].

The Forrest classification is the most frequently used bleeding ulcer classification system worldwide [19, 20]. The Forrest classification was used in 100% of the reported cases, both in HCV and LCV units. 

This inquiry shows a strong similarity in the proportion of ulcers according to the Forrest grades when comparing the data of HCV and LCV units. Ulcers with active bleeding (Forrest Ia, Ib) occurred in 23.8% in HCV units and in 22.1% in LCV units, whereas the proportion of ulcers with nonbleeding visible vessels (Forrest IIa) were almost identical (16.5% versus 16.6%).

Previous guidelines and conference reports recommended that all ulcers classified as Forrest Ia–IIb should be treated endoscopically [5, 14, 21]. A new meta-analysis by Laine and McQuaid revised the indication of endoscopic therapy in those Forrest IIb lesions with clots that resist vigorous rinsing [22]. By the modified recommendation, only patients with severe comorbidities should receive endoscopic therapy, and PPI therapy may be sufficient. 

In our survey, actively bleeding ulcers and ulcers with nonbleeding visible vessels were treated endoscopically with a surprisingly low frequency in LCV units because roughly one third of these cases were left untreated. Although these results are not much inferior to the France survey data [16], where Forrest IIa patients were endoscopically treated in 79%, we may draw the conclusion that mainly in LCV units either the technical facilities or the penetration of current guidelines are suboptimal.

The combination of haemostatic methods was used rather seldomly (34%) even in HCV units in our survey. Despite the fact that combination haemostatic therapy was used significantly more often in HCV units than in LCV units, one should conclude that for closing international standards, the combined haemostatic methods should be more promoted and also resourced [23].

According to current guidelines, intravenous bolus followed by continuous-infusion PPI should be used in patients with stigmata having undergone successful endoscopic therapy [5, 24, 25]. However, only 80% of the PUB cases were treated by i.v. PPI in our survey, which is a lower value compared to any other national inquiry on the same topic. Moreover, the best evidence 72-hour PPI administration policy [12, 13, 26] was followed in less than every other patient in both types of units. This deficit of a PPI treatment policy in our study may reflect the attitude of more focus on endoscopic therapy and less interest in medical treatment in the management of PUB patients.

When comparing HCV and LCV units, significant differences existed in some items of PUB management. In particular, haemostatic therapy with combination and PPI treatment by continuous infusion were used more frequently in those units with more PUB cases. Multivariate analysis showed that the only significant predictor factor for the use of these evidence-based standards of care was the monthly PUB case volume in the units. Some of these differences may be explained by poorer training or by less experience in lower case workplaces. Additionally, certain anomalies could be explained by the lack of financial resources in low case units, which mostly exist in smaller hospitals.

It is important to note that the financial reimbursement for haemostatic endoscopy has been rather low and inbalanced up to recently in Hungary which may be one of the reasons why in smaller hospitals the adherence to guidelines were suboptimal.

The main clinical outcome measures regarding the whole PUB cohort of this survey are comparable to previously published large databases [2, 27, 28]. In parallel with the trend that HCV units were closer to the best clinical practice consequences was seen on major clinical outcomes. Although rebleeding rates and the need for surgery were similar in the two units types, a significantly lower mortality rate was seen in HCV units compared to that in LCV units.

Considering these data, the question arises whether it is reasonable to manage PUB patients in LCV units or to centralize emergencies in HCV units is appropriate for many aspects like better offer of experienced endoscopiests and optimal utilization of financial sources are available.

Our study has some limitations similarly to other surveys dealing with PUB management in the daily routine. It is uncertain how has the registration influenced the results, and also there is no data whether the registration per se increased compliance with the guidelines. Although the inherent limitations of the survey, the comparison of different case volume workplaces adds some new knowledge.

In conclusion, our hypothesis that PUB case volumes have an effect on managing standards was mostly confirmed. Penetration of national recommendation and international guidelines is stronger in HCV units than in LCV units. Lower mortality of PUB patients in HCV units might be associated with better standards of care in those units. The results of this study could motivate medical societies and authorities to discuss whether PUB management needs further quality assurance efforts and better resources to improve daily practices.

## Figures and Tables

**Table 1:** Main points of the questionnaire for upper GI bleeders in Hungary.

(1) Structural and activity data	
(i) Practice of care (a) Gastroenterology unit (b) Surgical unit (c) Number of endoscopiests doing emergency endoscopy (ii) Number of bleeders per month (a) UGIB cases per month (b) PUB cases per month	

(2) Emergency endoscopy findings	
(i) Source of bleeding (ii) Number of ulcers and characteristics according to the Forrest classification	

(3) Endoscopic haemostatic therapy	
(i) Indication for endoscopic therapy (ii) Method of haemostatic therapy in different Forrest classes (a) Injection, substance of injection, mono; or in combination (b) Thermal (c) Clip (d) Combination therapy; components of combination	

(4) Acid-suppressant therapy in different Forrest classes	
(i) Substance for acid suppression; i.v. PPI or i.v. H_2_RA (ii) Method of i.v. PPI (a) Standard PPI dosage (b) Bolus + PPI infusion	

(5) Patient outcome data	
(i) Rebleeding rate (ii) Need for surgery (iii) Bleeding-related mortality	

**Table 2:** Comparison of selected items in high and low case volume units.

	HCV units (*n* = 23)	LCV units (*n* = 39)	*P* value
Number of PUB cases	1789	1244	not applicable
Mean number of PUB cases/endoscopists/month	3.2 ± 1.1	2.4 ± 0.9	ns

Item	% (*n*)	% (*n*)	

Endoscopic haemostatic treatment			
In Forrest Ia, Ib, and IIa	86 (613)	68 (327)	0.001
In Forrest IIb	45 (154)	39 (97)	0.015
Endoscopic treatment modality in Forrest Ia, Ib, and IIa			
Injection monotherapy	46 (285)	65 (311)	0.001
Haemoclip or thermocoagulation monotherapy	20 (121)	16 (51)	0.002
Combination	34 (207)	19 (61)	0.001
Acid-suppressant therapy after endoscopy with i.v. PPI			
Overall	79 (1413)	81 (1002)	0.490
In patients with Forrest I-II			
With standard dosages	28 (494)	48 (601)	0.001
With bolus + continuous PPI	50 (888)	33 (415)	0.001
Rebleeding rate	10.1 (179)	9.5 (118)	0.680
Need for surgery	5.1 (92)	6.4 (79)	0.181
Mortality			
Overall	2.7 (48)	4.3 (53)	0.023
In Forrest Ia, Ib	6.8 (29)	7.6 (21)	0.791

**Table 3:** Multiple logistic regression module of selected factors for the use of combined haemostatic methods followed by bolus plus continuous infusion of PPI in high-risk ulcer patients.

	Odds ratio	*P* value	95% CI
24-hour emergency endoscopy service available	0.82	0.738	0.92–1.12
Gastroenterology versus surgical unit	0.99	0.767	0.96–1.03
University versus municipal hospital	0.73	0.684	0.13–2.82
Mean number of endoscopiests per unit doing emergency endoscopy, <3 versus ≥3	2.28	0.078	0.91–5.76
Mean number of UGIB cases per month, <8.15 versus ≥8.15	5.48	0.012	1.88–18.42
